# From synaptic transmission to neural circuits: multilevel effects of stress on the hippocampus, bed nucleus of the stria terminalis, and paraventricular hypothalamic nucleus

**DOI:** 10.3389/fnsys.2026.1829622

**Published:** 2026-07-06

**Authors:** Hongye Zhou, Xinyi Wang, Sha Sha

**Affiliations:** 1 Department of Physiology, Nanjing Medical University, Nanjing, China; 2 Department of Stomatology, Nanjing Medical University, Nanjing, China; 3 The First Clinical Medical College, Nanjing Medical University, Nanjing, China

**Keywords:** bed nucleus of the stria terminalis, hippocampus, paraventricular hypothalamic nucleus, stress, synaptic function

## Abstract

The stress response is a non-specific adaptive physiological reaction that occurs when the organism is exposed to internal or external environmental stimuli, serving to maintain homeostatic balance. Its regulation involves highly complex neural mechanisms. The hippocampus, the bed nucleus of the stria terminalis (BNST), and the paraventricular hypothalamic nucleus (PVN) are key brain regions that play crucial roles in stress regulation. Focusing on the functional connectivity of the hippocampus-BNST-PVN neural circuit, this article systematically elucidates the multilevel effects of stress on synaptic transmission, synaptic plasticity, neural network integration, and related molecular mechanisms from microcosmic neurotransmitters to macroscopic neural circuits. These insights provide an important perspective for advancing our understanding of the neurobiological basis of stress and for developing new intervention strategies.

## 1 Introduction

Stress represents a critical adaptive response essential for maintaining physiological homeostasis, yet chronic or excessive stress exposure represents a major risk factor for widespread neuropsychiatric disorders, including anxiety, depression, post-traumatic stress disorder, and cognitive impairment. Mounting evidence indicates that stress profoundly reshapes brain structure, synaptic function, and neural circuit activity, particularly within limbic-hypothalamic networks that govern emotional processing and neuroendocrine output. Within this broader network, the hippocampus, bed nucleus of the stria terminalis (BNST), and paraventricular hypothalamic nucleus (PVN) constitute a key interconnected circuit involved in the sensing, integration, and coordination of stress responses through their regulation of the hypothalamic–pituitary–adrenal (HPA) axis and autonomic nervous system.

The hippocampus mediates contextual and cognitive modulation of stress and provides negative feedback control over the HPA axis. The BNST serves as a key integrative hub for sustained anxiety and autonomic arousal, relaying limbic signals to the PVN. As the final neuroendocrine output node, the PVN initiates stress hormone release and coordinates systemic adaptation. Despite extensive research on individual structures, a systematic synthesis of how stress acts across synaptic, cellular, and circuit levels within the hippocampus-BNST-PVN network remains incomplete.

This review focuses on the functional architecture and plastic changes of the hippocampus-BNST-PVN circuit under stress. We summarize anatomical connectivity, cellular subtypes, and synaptic transmission patterns, then outline stress-induced structural and functional plasticity, including dendritic remodeling, altered excitatory/inhibitory balance, and receptor-mediated molecular mechanisms. By integrating findings from synaptic transmission to neural circuit dynamics, this review aims to advance understanding of the neurobiological basis of stress and provide a theoretical foundation for identifying novel therapeutic targets for stress-related disorders.

## 2 The impact of stress on the organism

### 2.1 A general overview of stress

From a physiological perspective, stress is a systemic reaction triggered by the neuroendocrine system when the body encounters various internal and external environmental stimuli. Traditionally, stress was described as a non-specific response, suggesting that different stressors elicit a stereotyped physiological reaction involving activation of the hypothalamic–pituitary–adrenal (HPA) axis, stimulation of the sympathetic nervous system, and associated changes such as adrenal hypertrophy, thymic atrophy, and gastric ulceration. Nevertheless, this traditional definition represents an early simplified conceptualization and fails to integrate contemporary advances in stress neuroendocrinology. Cumulative modern evidence has revised this classical view, demonstrating that stress-induced neuroendocrine responses are not entirely non-specific. Instead, distinct stressor types can evoke unique neurochemical signatures, discrete physiological outputs, and differential recruitment of neural and neuroendocrine pathways (Agorastos and Chrousos, 2022). Generally, stressors can be categorized by their nature into physical stress from external environmental hazards, physiological stress derived from internal metabolic and homeostatic imbalance, and psychological–social stress induced by emotional and social challenges. Stress is also classified temporally into acute and chronic forms, which further diverge in neural circuit activation and endocrine profiling (McEwen, 2007).

The physiological mechanism of stress involves two systems: the sympathetic-adrenal medulla (SAM) system and the hypothalamic–pituitary–adrenal (HPA) axis. As an “emergency system,” the SAM system assists the body in achieving the “fight-or-flight” response when facing acute threats. Regulated by the autonomic nervous system (ANS), it secretes norepinephrine and adrenaline, and triggers a series of reactions in the cardiovascular, respiratory, renal, and endocrine systems.

The core neuroendocrine pathway of the stress response is the HPA axis (Tsigos and Chrousos, 2002). The PVN synthesizes and releases corticotropin-releasing hormone (CRH), which acts on CRH receptor type 1 (CRH-R1) in the anterior pituitary through the hypophyseal portal circulation to stimulate the release of adrenocorticotropic hormone (ACTH; Grammatopoulos and Chrousos, 2002). ACTH binds to the melanocortin 2 receptor (MC2R) on the surface of adrenal cells, stimulating the biosynthesis and secretion of glucocorticoids in the adrenal glands (Chida et al., 2007), regulating blood glucose levels, enhancing the sensitivity of the cardiovascular system to catecholamines, inhibiting inflammatory responses, thereby overcoming the interference of stressors on homeostasis.

The stress-activated SAM and HPA systems coordinate rapid and long-term responses to stress. SAM activation mediates acute “fight-or-flight” responses and induces persistent transcriptional changes in central and peripheral tissues, modulating adrenergic receptor expression, intracellular signaling, and gene networks involved in synaptic plasticity (Kvetnansky et al., 2009). The HPA axis is generally considered to have a slower onset but longer duration, which can maintain the stability of the internal environment of the human body and induce physiological responses to adapt to external stimuli (Rasiah et al., 2023). However, glucocorticoids and mineralocorticoids from the HPA axis exert classical genomic effects via intracellular steroid receptors, producing sustained changes in synaptic function, neurogenesis, and stress reactivity, while also triggering rapid non-genomic actions that modulate neuronal excitability, synaptic transmission, and feedback within minutes (Joëls et al., 2008; Tasker and Herman, 2011). Both systems converge on hippocampal, BNST, and PVN circuits, shaping behavioral and physiological outcomes, and chronic dysregulation contributes to anxiety, hyperarousal, cognitive impairment, cardiovascular strain, and metabolic disturbances.

### 2.2 Effects of stress on systemic physiological functions

Stress exerts dual effects on the body: moderate stress promotes adaptation, whereas intense or prolonged stress disrupts physiological homeostasis (McEwen, 2007). Acute stress activates the HPA axis, releasing cortisol, which mobilizes energy, suppresses non-essential systems, modulates immune metabolism via itaconate (Auger et al., 2024), inhibits reproduction through RFRP-3-mediated GnRH suppression (Rasiah et al., 2023; Mamgain et al., 2021), and regulates cardiovascular and inflammatory responses while providing anti-allergic, anti-toxic, and anti-shock effects (Black and Garbutt, 2002).

Stress-induced dysregulation of the SAM system and HPA axis can activate the renin-angiotensin-aldosterone system (RAAS), as catecholamines and ACTH stimulate RAAS, with ACTH also promoting aldosterone synthesis and synergizing with RAAS to regulate homeostasis. Chronic co-activation of these pathways may result in persistent hypertension, vascular remodeling, renal strain, and metabolic disturbances (Correa et al., 2022; Golbidi et al., 2015; Terock et al., 2019). Acute stress activates the sympathetic nervous system and HPA axis, increasing cardiovascular output, redistributing blood, and enhancing brain stress sensitivity (do Vale et al., 2020; Joyner and Casey, 2015; Kim and Kim, 2023), while adrenaline via *β*-adrenergic signaling and angiotensin II drive cardiac inflammation, hypertrophy, fibrosis, and mitochondrial dysfunction (Dang et al., 2020; Liu et al., 2022, 2023). Chronic stress sustains these pathways, promoting water and sodium retention, hypertension, remodeling, and heart failure (Golbidi et al., 2015).

Acute stress transiently enhances immunity by promoting inflammatory factors, activating natural killer cells and macrophages, and recruiting white blood cells via corticosterone and adrenaline (Alotiby, 2004), whereas chronic stress impairs immune function, inhibits lymphocyte proliferation, disrupts cytokine balance, and, through prolonged glucocorticoid exposure, induces GR desensitization, hippocampal dendritic atrophy, and deficits in memory and emotional regulation (Dhabhar, 2014; Kim and Kim, 2023).

In summary, acute stress activates the HPA axis and sympathetic nervous system, releasing cortisol, adrenaline, and norepinephrine to enhance cardiovascular output, glucose availability, respiration, and muscle perfusion, facilitating adaptive responses to immediate threats. Chronic stress, however, sustains these hormonal and sympathetic activations, leading to impairments in cardiovascular function, cognition, emotional regulation, and other systems, producing widespread negative effects.

### 2.3 Effects of stress on the structure and function of the nervous system

#### 2.3.1 Effects of stress on the central nervous system

The response of the nervous system to stress depends on the interaction between the central nervous system (CNS) and the ANS. Correspondingly, both acute and chronic stress exert varying degrees of influence on the structure and function of the nervous system. Studies have shown that stress affects the CNS through multiple aspects, including neuronal plasticity, neuroendocrinology, neuroinflammation, and epigenetics (Yuan et al., 2023). Relevant hormones or neuroendocrine factors produced by stress act on the nervous system, leading to changes in the structure and density of neural networks, thereby affecting neural function and altering brain structure. Research indicates that steroids, glutamate, and brain-derived neurotrophic factor (BDNF) can all mediate changes in neuronal structure. For example, glutamate and NMDA receptors in the prefrontal cortex (PFC) are involved in the process of dendritic proliferation and atrophy induced by stress, and inhibiting NMDA receptors can block dendritic remodeling caused by chronic stress (McEwen et al., 2016).

Studies investigating neural plasticity demonstrate that chronic stress triggers dendritic atrophy and suppressed neuronal activity within the hippocampus and prefrontal cortex (PFC), whereas it facilitates dendritic proliferation and heightened neuronal firing in the amygdala (McEwen et al., 2016; Roozendaal et al., 2009). As a core brain region governing affective processing, the amygdala primarily mediates fear and anxiety modulation. Acute stress transiently elevates amygdala activity to facilitate rapid emergency coping; nevertheless, persistent chronic stress drives pathological amygdalar overactivation, which further precipitates anxiety- and fear-related affective disorders (Zhang et al., 2021). In addition, the PFC is closely related to emotional regulation and self-control ability (van de Poll et al., 2023; Awasthi et al., 2020). Chronic stress-induced PFC atrophy impairs its top-down regulatory capacity over emotion and behavioral output, rendering individuals prone to impulsivity and affective lability under stressful challenges. On the other hand, the hippocampus is an essential component of the negative feedback loop of the HPA axis. Stress downregulates the function of the hippocampus, resulting in negative feedback inhibition of the HPA axis (Mika et al., 2012; Cole et al., 2022). Sustained activation of the HPA axis can disrupt the rhythm of cortisol secretion and even cause exhaustion of HPA axis function (Herman et al., 2016).

Beyond neurons, stress also induces glial-cell-mediated neuroinflammation. When peripheral inflammatory stimuli arise, circulating cytokines cross the blood–brain barrier or relay inflammatory signals to the CNS through vagal afferent pathways (Garcia-Dominguez, 2026). This triggers microglial release of inflammation-related cytokines, which diffuse through cerebrospinal fluid or interstitial fluid in the brain, subsequently activating indoleamine 2,3-dioxygenase (IDO). IDO-dependent tryptophan metabolites act as NMDA receptor agonists, enhancing glutamatergic synaptic transmission and inducing depressive-like behaviors (Walker et al., 2014). Finally, chronic stress can produce long-lasting effects on brain function by altering gene expression through epigenetic modifications. Evidence from early-life adversity studies shows that early stress induces DNA methylation of exon 17 of the glucocorticoid receptor (GR) promoter region in rats, as well as the exon 1F variant of the human GR gene, leading to increased HPA axis sensitivity to stress (Turecki and Meaney, 2016).

#### 2.3.2 Effects of stress on the peripheral nervous system and emotional regulation

Stress also exerts multiple effects on the peripheral nervous system. First, stress can cause autonomic imbalance, in which continuous activation of the sympathetic nervous system *via* the SAM axis and inhibition of the parasympathetic system (particularly the vagus nerve) result in elevated resting heart rate, reduced heart rate variability (HRV), and increased baseline blood pressure (Thayer and Lane, 2009; Bonaz et al., 2017). Second, stress negatively regulates the enteric nervous system and alters gut permeability and microbiota composition through the brain–gut axis, thereby affecting gastrointestinal function—providing the neural basis for stress-related digestive disturbances (Mayer et al., 2014). Finally, chronic stress can lower pain thresholds, amplify pain perception, and lead to chronic widespread pain (such as fibromyalgia). The mechanism is associated with central sensitization (e.g., increased excitability of dorsal horn neurons) and dysfunction in descending pain-modulation pathways (Jennings et al., 2014).

Although stress can improve the brain’s sensitivity and attention to emotional stimuli, it can also impair emotional regulation ability, cause negative emotions such as anxiety and depression, and even lead to stress-related emotional disorders. Positive emotional regulation is often accompanied by activation of the prefrontal cortex and hippocampus and decreased activation of the amygdala. In contrast, stress-exposed subjects often show enhanced activation of the amygdala and weakened activation of the prefrontal cortex and hippocampus. On this basis, chronic stress (including early-life stress) often leads individuals to adopt negative regulation strategies such as suppression and avoidance in daily life, and these two strategies have a strong positive correlation with psychological disorders such as depression and anxiety (Conduah et al., 2025). Similarly, chronic variable stress (CVS), a classic preclinical rodent model featuring intermittent exposure to diverse unpredictable mild stressors, drives animals subjected to recurrent unavoidable physical, social and psychological challenges to develop robust depressive-like behavioral phenotypes (Rasiah et al., 2023).

Studies indicate that the effects of stress on the nervous system and their underlying mechanisms are associated with abnormal glial-cell function (Rana et al., 2021). Under chronic stress, dopaminergic projections from the ventral tegmental area (VTA) to the nucleus accumbens (NAc) are diminished in depressed individuals, reducing the experience and motivation related to reward, leading to anhedonia. Impairment of astrocytic function can cause abnormal glutamatergic signaling in the NAc. In the brains of patients with stress-induced depression, astrocytes show structural atrophy, reduced numbers, and functional dysregulation, weakening their support for neurons and leading to neuronal atrophy and loss, together with reduced release of ATP which result in purinergic-signaling imbalance. In addition, it has been found that reductions in glutamate transporter (GLT-1) levels among the patients impair glutamate clearance, increasing synaptic glutamate concentrations, which further contributes to PFC and hippocampal volume loss and ultimately produces depressive-like behaviors (Cao et al., 2013; Sanacora et al., 2012; Tynan et al., 2013). Microglia, the resident immune cells of the brain, release pro-inflammatory cytokines (such as IL-1β and TNF-*α*) upon stress activation. These cytokines suppress BDNF expression, disrupt monoamine metabolism, impair neuroplasticity, and directly induce sickness behaviors such as anhedonia and social withdrawal (Wohleb et al., 2016).

## 3 Structure and function of the hippocampus, BNST and PVN

### 3.1 Hippocampus: anatomy and cellular components

The hippocampus is part of the temporal lobe system and is located beneath the cerebral cortex, along the inferior horn and medial wall of the lateral ventricle. Its anterior end is enlarged and called the hippocampal foot, the upper part is prominent as the hippocampal digit, and the posterior end is deeply curled with the hippocampal fissure, and the lower wall of the hippocampal fissure forms the hippocampal gyrus (Schultz and Engelhardt, 2014).

Anatomically, the human hippocampus is divided into four sectorial subregions—CA1, CA2, CA3, and CA4—based on cellular morphology and cortical development, with each subregion performing distinct cognitive functions. CA1 plays a critical role in memory processes; CA2 is involved in the storage and encoding of spatial information; and CA3 is responsible for associating stimulus–pattern matching with prior experiences (Cherubini and Miles, 2015; Chauhan et al., 2021). Across humans and laboratory rodents, the hippocampal formation displays a highly conserved but subfield-specific laminar organization. Although the alveus, stratum oriens, stratum pyramidale, stratum radiatum, and stratum lacunosum-moleculare are common features across most hippocampal subfields, the stratum lucidum is largely confined to CA3, with a less prominent presence in CA2, and is absent from CA1 and the subiculum. At the cellular level across mammalian species, hippocampal neurons are grouped into two principal classes: glutamatergic principal pyramidal neurons and heterogeneous non-principal interneurons. Soma of nearly all pyramidal neurons resides within the stratum pyramidale. In contrast to the relatively well-defined principal excitatory neuron populations of the hippocampus, GABAergic interneurons display prominent subtype diversification, encompassing basket cells, bistratified cells, and oriens lacunosum moleculare (O-LM) interneurons, which mediate distinct inhibitory patterns in hippocampal microcircuits. Specifically, basket cells serve as perisomatic-targeting interneurons that regulate the overall firing of pyramidal neurons, while bistratified cells and O-LM cells are typical dendrite-targeting interneurons that modulate dendritic synaptic integration (Chauhan et al., 2021; Graves et al., 2012; Müller and Remy, 2014). Collectively, these various neuronal types form intricate microcircuits via compartment-specific inhibition: perisomatic and dendritic-targeted interneurons mediate feedforward and feedback inhibitory loops to tune pyramidal cell excitability, thereby regulating synaptic plasticity for memory formation and storage (Müller and Remy, 2014).

### 3.2 BNST: subregions and neuronal characteristics

The bed nucleus of the stria terminalis (BNST) is located in the basal forebrain and can be divided into multiple subregions according to cell structure, neurochemistry, and connection patterns. In rodents, the bed nucleus of the stria terminalis (BNST) is anatomically subdivided into dorsal and ventral regions. The dorsal part mainly includes the dorsal BNST (dBNST) and dorsolateral BNST (dlBNST), which are rich in CRH-expressing neurons. The ventral part mainly includes the ventral BNST (vBNST), ventrolateral BNST (vlBNST), and oval nucleus BNST (ovBNST), which are rich in neurons expressing neuropeptides such as Vasopressin and Orexin, and are closely related to reproductive behavior, social behavior, and reward systems (Dong and Swanson, 2001). Unlike in rodents, human BNST subdivisions are less clearly demarcated at the microscopic level due to limitations in imaging and histology. Based on postmortem and neuroimaging studies, the BNST in humans is commonly described as comprising anterolateral, anteromedial, and posterior divisions, each containing heterogeneous populations of neurons, including GABAergic and peptidergic cells. The human BNST is involved in stress and anxiety regulation, autonomic control, and integration of emotional and social information, paralleling to functions observed in rodent BNST subregions (Avery et al., 2016).

Based on neurotransmitter synthesis, BNST neurons can be classified into glutamatergic neurons and GABAergic neurons, and the relative proportions of these neuron types vary across subregions. The majority of BNST neurons are GABAergic (∼97%) with a very small glutamatergic population (∼3%; Kudo et al., 2012), among these, the vBNST contains the highest number of glutamatergic neurons; in the vlBNST, approximately 80% of neurons are GABAergic projection neurons or local interneurons, while only about 20% are glutamatergic projection neurons (Ortiz-Juza et al., 2021). Additionally, neurons in different BNST subregions display considerable morphological diversity. A study in rats showed that in the juxtacapsular nucleus of the BNST, most neurons are small, spiny, and predominantly bipolar. In the ovBNST, 10 additional neuronal types have been identified, including oval-shaped, fusiform, and polygonal neurons, which may include both projection neurons and local interneurons. In the human BNST, various morphologies have also been observed, including fusiform, triangular, medium-sized, and small basket-like neurons. Compared with rodents, neurons in the anterolateral BNST of primate exhibit longer dendritic lengths and greater dendritic branching, suggesting that individual neurons receive a broader diversity of input signals (van de Poll et al., 2023).

### 3.3 PVN: organization and functional regulation

The paraventricular nucleus of the hypothalamus (PVN), located dorsolateral to the third ventricle, is a central hub for neuroendocrine and autonomic regulation in both rodents and humans. In rodents, PVN neurons are classically divided into magnocellular and parvocellular populations. Magnocellular neurons synthesize oxytocin (OT) and vasopressin (VP), projecting to the posterior pituitary for systemic hormone release, while collateral projections reach extrahypothalamic targets, including the hippocampus, amygdala, nucleus accumbens, lateral septum, and locus coeruleus, influencing stress, reward, and social behaviors. Parvocellular neurons modulate autonomic output and anterior pituitary function via projections to the median eminence and brainstem. In humans, PVN neurons display a similar magnocellular/parvocellular organization, with magnocellular cells releasing OT and VP systemically and parvocellular cells regulating neuroendocrine and autonomic processes. However, direct extrahypothalamic projections are less well characterized in humans, and functional evidence largely derives from neuroimaging and postmortem studies. Despite these anatomical differences, the human PVN similarly contributes to stress regulation, autonomic homeostasis, fluid balance, and social–emotional processing (Harding et al., 1995; Goel et al., 2024).

Parvocellular neurons are further divided into parvocellular neurosecretory cells (PNCs) and non-neurosecretory parvocellular preautonomic neurons (PANs). PNCs synthesize hypophysiotropic hormones—including corticotropin-releasing hormone (CRH), thyrotropin-releasing hormone (TRH), gonadotropin-releasing hormone (GnRH), and somatostatin—and project to the median eminence, where they release these hormones into the hypophyseal portal system. PANs, on the other hand, project primarily to autonomic centers in the brainstem and spinal cord to regulate sympathetic and parasympathetic output. These target regions include the nucleus tractus solitarii (NTS), the rostral ventrolateral medulla (RVLM), and the intermediolateral column (IML) of the spinal cord. In the rat brain, magnocellular neuron somata are located laterally within the PVN, while parvocellular neuron somata are located medially; in the mouse brain, this arrangement is reversed. The dendrites of these neuronal populations do not remain segregated but intermingle extensively, enabling multiple modes of intra-nuclear communication (Rasiah et al., 2023).

## 4 Effects of stress on the hippocampus-BNST-PVN neural circuit and the underlying mechanisms

### 4.1 The hippocampus-BNST-PVN neural circuit

#### 4.1.1 Direct projections and regulatory logic of the core circuit

Research on rodents has found that forebrain limbic regions such as the hippocampus, amygdala, and medial prefrontal cortex project nerve fibers to basal forebrain regions such as the BNST or internal hypothalamic regions, which in turn send projections to the PVN to regulate its function. On the one hand, the hippocampus, BNST, and PVN participate in mediating endocrine stress responses by regulating the function of the HPA axis (Myers et al., 2013). Downstream of the BNST, within the anterior BNST, corticotropin-releasing hormone (CRH) neurons in subregions such as the dorsomedial and fusiform nuclei project to the medial parvocellular region of the PVN (PVNmp). GABAergic neurons in the dorsomedial and magnocellular nuclei project to the PVN to inhibit the hypothalamic activity, while glutamatergic neuron populations in the anteroventral nucleus exert excitatory effects on the PVN (Maita et al., 2022). In the posterior BNST, GABAergic neuron populations in the principal nucleus also project to the PVNmp, participating in suppressing HPA axis responses to stress. Notably, rodents share highly conserved brain structural and functional neural connectivity with humans, especially in the above-mentioned limbic-BNST-hypothalamic-PVN circuit, which provides a solid anatomical foundation for applying rodent models to explore the neural regulatory mechanism of HPA axis activity (Xu et al., 2022).

Upstream, the principal nucleus of the BNST receives extensive inputs from multiple limbic regions. For example, the medial amygdala excites the HPA axis through GABA-GABA disynaptic inhibition at the level of the PVN, whereas stimulation of the hippocampus (ventral subiculum) inhibits the principal nucleus, thereby suppressing PVN activity and inhibiting the HPA axis (Choi et al., 2007; Cole et al., 2022). On the other hand, the BNST also regulates physiological stress responses through its connection with the PVN. The BNST is associated with descending autonomic outputs and ascending visceral sensory inputs. The oval nucleus of the dorsal BNST is involved in pre-autonomic signal transmission, while the ventral BNST receives dense noradrenergic signals from the brainstem nuclei of the nucleus tractus solitarius and the ventrolateral medulla, and is primarily responsible for visceral sensory processing. This ventral BNST subnucleus (fu/av) mainly projects directly and densely to the PVN via CRH neurons, thereby modulating physiological responses to stress (Sibbach et al., 2023; Myers et al., 2013).

#### 4.1.2 Extra-limbic connections and brainstem inputs to the PVN

In addition to the aforementioned neural circuits, there are extensive connections between the BNST and other brain regions. The ventral BNST innervates the ventral tegmental area (VTA), and stimulating GABAergic projections can inhibit VTA-GABAergic neurons, leading to anxiolytic-like behaviors, while stimulating glutamatergic projections results in anxiogenic behaviors in mice (Jennings et al., 2013). The input and output regions of the anterolateral BNST are similar. The primary outputs related to stress and anxiety functions include specific regions of the cerebral cortex, amygdala, hypothalamus and thalamus, basal ganglia, and midbrain/hindbrain autonomic centers, which include GABAergic and glutamatergic projections. Among the input sites, the basolateral amygdala (BLA), PFC, hippocampus, and PVN mainly provide glutamatergic inputs, while the central amygdala (CeA), medial amygdala (MeA), and NAc mainly provide GABAergic inputs (van de Poll et al., 2023).

The relay sites for indirect projections from forebrain limbic regions to the PVN include not only the BNST but also the dorsomedial hypothalamus (DMH) and medial preoptic area (mPOA), both of which provide predominantly inhibitory inputs to the PVN (Myers et al., 2013). In addition to these forebrain pathways, the PVN receives substantial catecholaminergic innervation from medullary cell groups, particularly those within the nucleus of the solitary tract (NTS) and ventrolateral medulla. The anatomically distinct parabrachial nucleus (PBN), which maintains reciprocal connections with the NTS, contributes primarily non-catecholaminergic signaling. Together with the DMH and BNST, the PBN represents an important component of the excitatory network regulating PVN CRH neuron activity and HPA axis responses to stress (Kaur et al., 2013; Ulrich-Lai and Herman, 2009). In addition, the median raphe nucleus and dorsal raphe nucleus provide serotonergic projections to CRH neurons in the PVN. Activation of 5-HT2C receptors stimulates PVN CRH and increases ACTH release. Three circumventricular organs (CVOs), the subfornical organ (SFO), organum vasculosum of the lamina terminalis, and area postrema, detect chemical inputs in the systemic circulation and project to the PVN (Ulrich-Lai and Herman, 2009). The CRH neurons in the PVN mainly project to the median eminence to initiate neuroendocrine responses (Rasiah et al., 2023). Overall, the hippocampus-BNST-PVN circuit represents a highly interconnected network through which limbic and hypothalamic regions converge to modulate both neuronal excitability and endocrine output under stress.

### 4.2 Stress-induced plasticity and molecular mechanisms in the Hippocampus-BNST-PVN circuit

#### 4.2.1 Stress-induced structural and functional plasticity in the hippocampus

The hippocampus-BNST-PVN circuit, a central pathway mediating stress responses, is largely driven by projections from the subiculum, the principal output region of the hippocampus. The ventral subiculum, in particular, serves as a major conduit for conveying hippocampal information to limbic and hypothalamic structures, including the BNST and hypothalamic nuclei that regulate HPA axis activity (O’Mara, 2006; Lee et al., 2019). Functionally, the hippocampus exhibits a pronounced dorsal–ventral specialization: the ventral hippocampus is primarily involved in emotional and stress-related processing, whereas dorsal regions contribute more to spatial and mnemonic functions (O’Mara, 2006; Kim et al., 2015). Within the ventral hippocampus, diverse pyramidal neuron subtypes—including burst-spiking pyramidal cells—are differentially distributed along the proximal–distal axis from CA1 into the subiculum, contributing to output specificity (Kim and Spruston, 2012). Burst-firing neurons are more prevalent in the distal subiculum, supporting specialized encoding and transmission of stress-related signals to downstream limbic and reward circuits (Jarsky et al., 2008). Chronic stress paradigms, such as social defeat, selectively increase the proportion of strongly bursting neurons in the ventral subiculum, linking stress exposure to altered firing patterns in hippocampal outputs (Lee et al., 2019).

In addition to these subregion- and cell-type-specific features, the hippocampus exhibits pronounced plasticity in response to stress. Chronic stress induces dendritic atrophy in CA3 and dentate gyrus neurons and spine loss in CA1 pyramidal cells, while restraint and multimodal stress reduce synapse numbers in CA3 (Christian et al., 2011; Magariños et al., 1997); multimodal stress alone significantly diminishes dorsal CA1 synapses and alters connectivity, weakening projections to the septum and thalamus while enhancing amygdala and BNST connections (Maras et al., 2014; McEwen et al., 2016). Acute and chronic stress can also inhibit hippocampal neurogenesis and reduce newborn cell survival via excessive HPA axis activation and elevated circulating glucocorticoids; these detrimental outcomes are partly mediated by functional changes in dentate granule neurons coupled with aberrant NMDA receptor signaling. In contrast, environmental and behavioral interventions including physical exercise and social enrichment effectively reverse stress-induced impairments by boosting the synthesis and secretion of neurotrophic factors (Parul et al., 2021; Schoenfeld and Gould, 2012). Functionally, stress impairs long-term potentiation (LTP) and enhances long-term depression (LTD), leading to deficits in synaptic plasticity, learning, and memory (Kim and Diamond, 2002). Together, these findings indicate that stress regulation is not uniform across the hippocampus but relies on specialized ventral subregional outputs, specific cell types, and dynamic structural and functional plasticity, forming a central node within the hippocampus–BNST–PVN stress circuitry.

#### 4.2.2 Stress-induced plasticity in the BNST and PVN

In contrast to the hippocampus, chronic stress typically enhances dendritic spine proliferation and neuronal excitability in the BNST. Chronic restraint stress increases BNST volume and dendritic arborization, promoting avoidance behavior in male rats (McEwen, 2007; Pêgo et al., 2008), and various chronic stress models upregulate glutamate, GABA, and NMDA receptors, altering postsynaptic responses and synaptic efficacy (Maita et al., 2022). The ovBNST, composed predominantly of CRF-positive GABAergic neurons, exhibits increased excitability under stress through modifications of LTP firing rates, postsynaptic currents, and resting membrane potentials via PKA and PACAP pathways, leading to persistent CRF expression and changes in CRFR expression. These adaptations enhance inhibitory projections to the anterodorsal BNST, disinhibiting the PVN and activating the HPA axis (Hu et al., 2020; Maita et al., 2022).

The PVN itself exhibits both neuroendocrine and synaptic plasticity under stress. Acute stress increases VP mRNA expression, and VP acts as an ACTH secretagogue to activate the HPA axis and stimulate corticosterone release (Bartanusz et al., 1993). Corticosterone, in turn, exerts negative feedback by modulating PVN neuron excitability and CRH synthesis through alterations in membrane currents, endocannabinoid (eCB), and CB1R-mediated presynaptic inhibition (Kim et al., 2019). Stress dynamically modulates PVN synapses: acute stress induces short-term potentiation in glutamatergic synapses and LTP in GABAergic synapses, whereas elevated corticosterone promotes LTD through enkephalin-mediated *μ*-opioid receptor signaling (Dos-Santos et al., 2023; Grammatopoulos and Chrousos, 2002; Yamaguchi et al., 2024). Chronic stress increases glutamatergic input and weakens GABAergic inhibition in PVN CRH neurons, elevating excitatory drive and impairing HPA axis negative feedback (Bains et al., 2015; Rasiah et al., 2023). Early life stress further primes CRH neurons toward spontaneous activity, independent of excitatory input (Rasiah et al., 2023).

#### 4.2.3 Corticosteroid and neurotransmitter receptor-mediated mechanisms

Hippocampal and hypothalamic corticosteroid receptors mediate both basal and stress-induced HPA axis activity. Mineralocorticoid receptors (MR) in the hippocampus stabilize CA1 excitability, while glucocorticoid receptors (GR) inhibit CA1 excitatory output during acute stress and regulate HPA negative feedback in hypothalamic CRH neurons. Cortisol exerts its effects through both genomic and non-genomic GR signaling pathways. Rapid non-genomic GR activation can engage eCB signaling, which suppresses presynaptic glutamate release and contributes to stress-related synaptic modulation (Di et al., 2003). In contrast, chronic stress and HPA axis dysregulation have been associated with reduced GR expression and impaired glucocorticoid feedback sensitivity in PVN neurons (Herman et al., 2016; Rasiah et al., 2023).

Glutamate and GABA receptors further shape synaptic plasticity. Ionotropic (NMDAR, AMPAR, KAR) and metabotropic (mGluRs) glutamate receptors mediate fast and slow excitatory signaling, respectively, and acute stress increases NMDAR/AMPAR ratios in PVN parvocellular neurons, enhancing synaptic sensitivity (Reiner and Levitz, 2018; Kuzmiski et al., 2010). Chronic stress upregulates NMDA receptors in the BNST, increases glutamatergic PVN inputs, and impairs eCB-mediated inhibition, leading to excessive HPA axis activation (Maita et al., 2022). GABA_A_ and GABA_B_ receptors mediate inhibitory tone; chronic stress alters GABA_A_ receptor subunit composition and functional synapse distribution in the BNST and PVN, reducing inhibitory efficacy despite increased total synapse number (Gao et al., 2017; Hewitt et al., 2009; Maita et al., 2022).

#### 4.2.4 Sexually dimorphic effects of stress on the hippocampus-BNST circuit

Importantly, stress exerts pronounced sexually dimorphic effects on the brain, particularly within the hippocampus–BNST circuit. Both regions exhibit sex-dependent differences in synaptic physiology, connectivity, and stress responsiveness. For instance, male and female rodents display divergent patterns of dendritic remodeling, spine density changes, and excitatory/inhibitory synaptic balance in response to chronic stress, which may contribute to sex-specific behavioral outcomes such as anxiety, fear processing, and stress coping (Bangasser and Valentino, 2014; McCarthy et al., 2012). Moreover, the BNST shows marked sexual dimorphism in size, neuronal composition, and hormone receptor expression, which underlies its differential regulation of stress and autonomic circuits in males versus females (Crestani et al., 2013; Chung et al., 2002).

## 5 Summary

**Figure 1:**
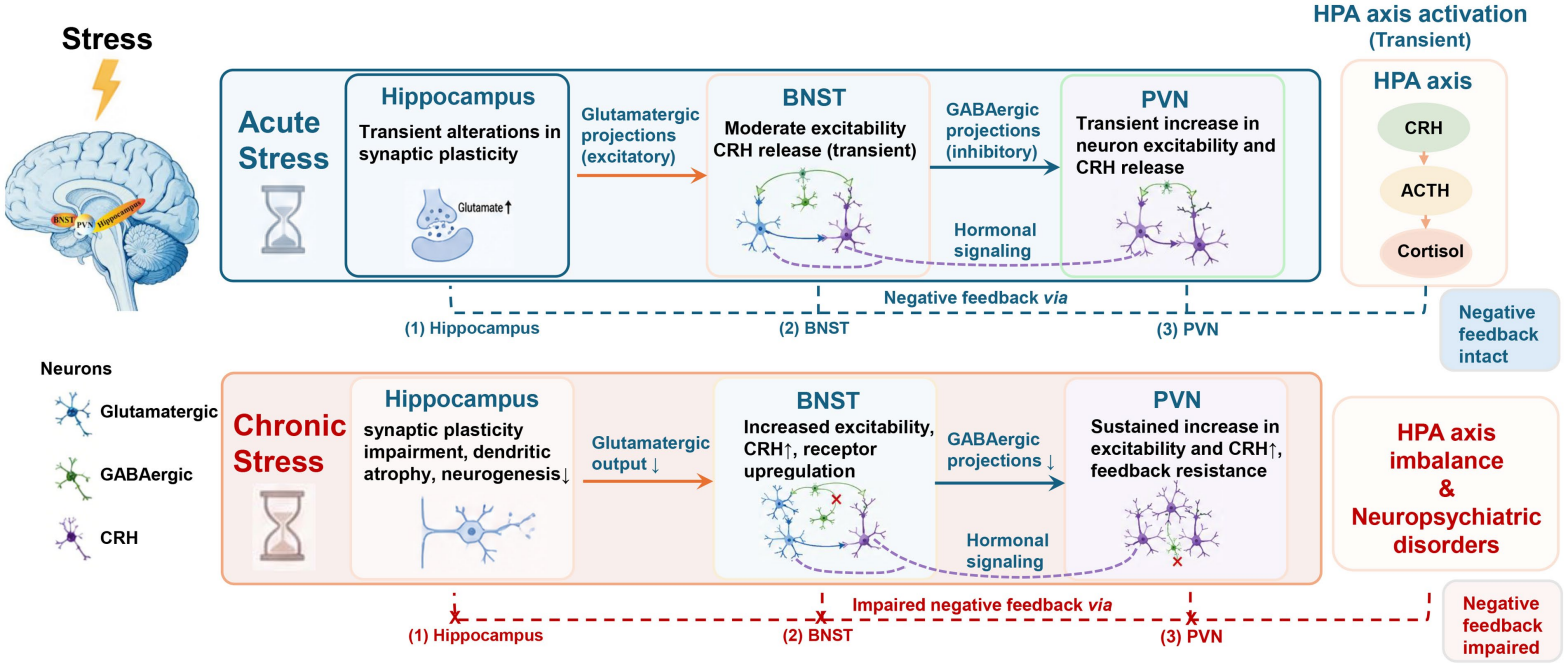
Differential effects of acute and chronic stress on the hippocampus-BNST-PVN circuit and HPA axis regulation. Schematic illustration of the neural and endocrine adaptations induced by acute and chronic stress. Under acute stress, transient alterations in hippocampal synaptic plasticity, moderate activation of the bed nucleus of the stria terminalis (BNST), and a temporary increase in paraventricular nucleus (PVN) excitability promote corticotropin-releasing hormone (CRH) release and transient activation of the hypothalamic–pituitary–adrenal (HPA) axis, resulting in increased adrenocorticotropic hormone (ACTH) and cortisol secretion. Intact negative-feedback regulation mediated by the hippocampus, BNST, and PVN subsequently restores HPA axis homeostasis. In contrast, chronic stress induces persistent hippocampal synaptic dysfunction, dendritic atrophy, and reduced neurogenesis, accompanied by increased BNST excitability, elevated CRH signaling, receptor upregulation, and sustained PVN hyperexcitability with feedback resistance. Reduced hippocampal glutamatergic output and impaired BNST-mediated GABAergic inhibition weaken negative-feedback control of the HPA axis, leading to prolonged CRH secretion, HPA axis dysregulation, and increased vulnerability to neuropsychiatric disorders.

Accumulating evidence suggests that stress-induced alterations in hippocampal, BNST, and PVN circuits contribute to both neuropsychiatric and systemic consequences of chronic stress. Changes observed across these regions, including impaired hippocampal synaptic plasticity, altered BNST excitability, and dysregulated CRH signaling, are associated with disruptions in HPA-axis regulation. During acute stress, PVN neuronal activity is transiently enhanced and subsequently constrained by glucocorticoid-mediated negative feedback, whereas chronic stress is frequently associated with prolonged PVN activation and impaired feedback regulation, although the magnitude and persistence of these effects depend on the stress paradigm and experimental context. Available data support the view that such circuit-level adaptations contribute to stress-related phenotypes, including anxiety, depression, and PTSD (Figure 1) (Lebow and Chen, 2016; Zheng et al., 2026).

Evidence from both clinical and preclinical studies further suggests that stress-related disorders are accompanied by distinct, but partially overlapping, alterations in HPA-axis function. In PTSD, altered cortisol dynamics and stress responsivity have been associated with changes in BNST activity and HPA-axis regulation, although the direction and magnitude of these effects vary across studies (Awasthi et al., 2020; Lebow and Chen, 2016; Petranu et al., 2024). In major depressive disorder, hippocampal atrophy and impaired hippocampal regulation of stress-responsive circuits have been linked to enhanced HPA-axis activity in some patient populations and experimental models (Cole et al., 2022; Grace and Uliana, 2026). Chronic disturbances in glucocorticoid signaling are also associated with systemic consequences, including altered adipose tissue distribution, hair growth abnormalities, and reduced bone density (Choi et al., 2021; Epel et al., 2000; Weinstein et al., 1998).

Importantly, pharmacological interventions targeting stress-related neuroendocrine pathways, such as CRHR1 antagonists, have shown potential to alleviate affective symptoms in preclinical studies. In contrast, the beneficial effects of dapagliflozin may involve broader metabolic, inflammatory, and neuroendocrine mechanisms rather than direct normalization of CRH or glucocorticoid signaling (Wu and Zetter, 2022). Together, these findings are consistent with the view that stress-induced synaptic and circuit remodeling contributes to the pathophysiology of stress-related disorders and may represent a target for therapeutic intervention.
